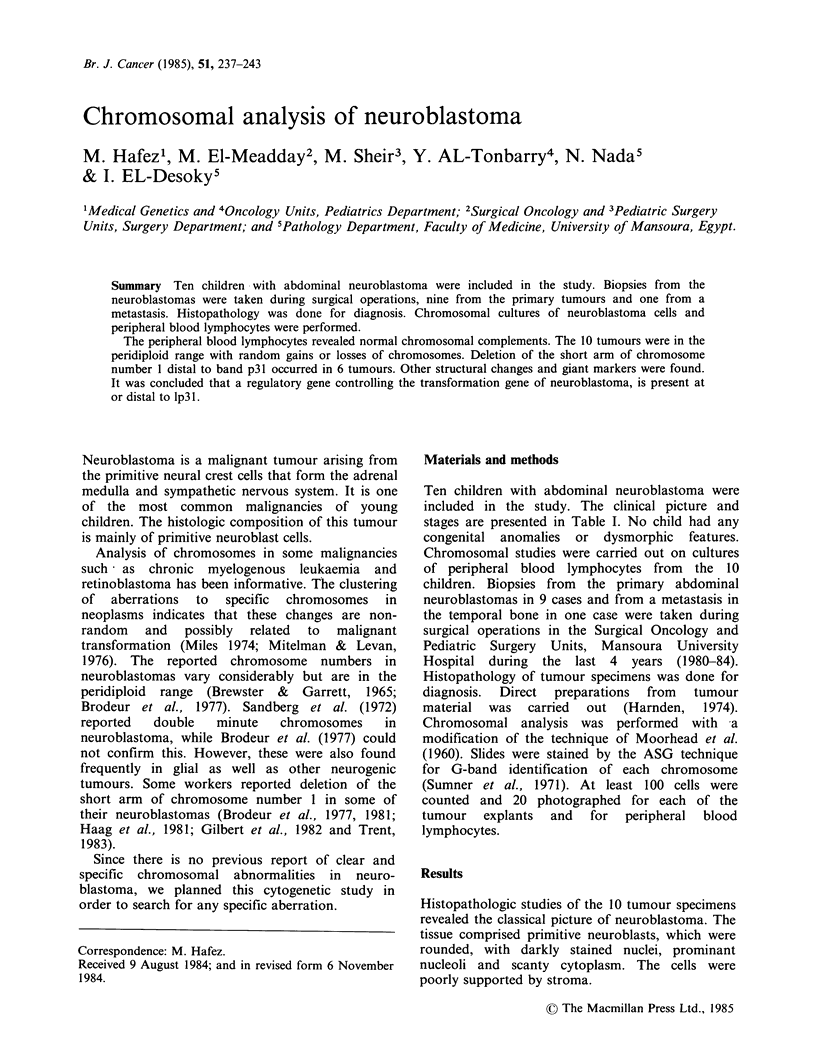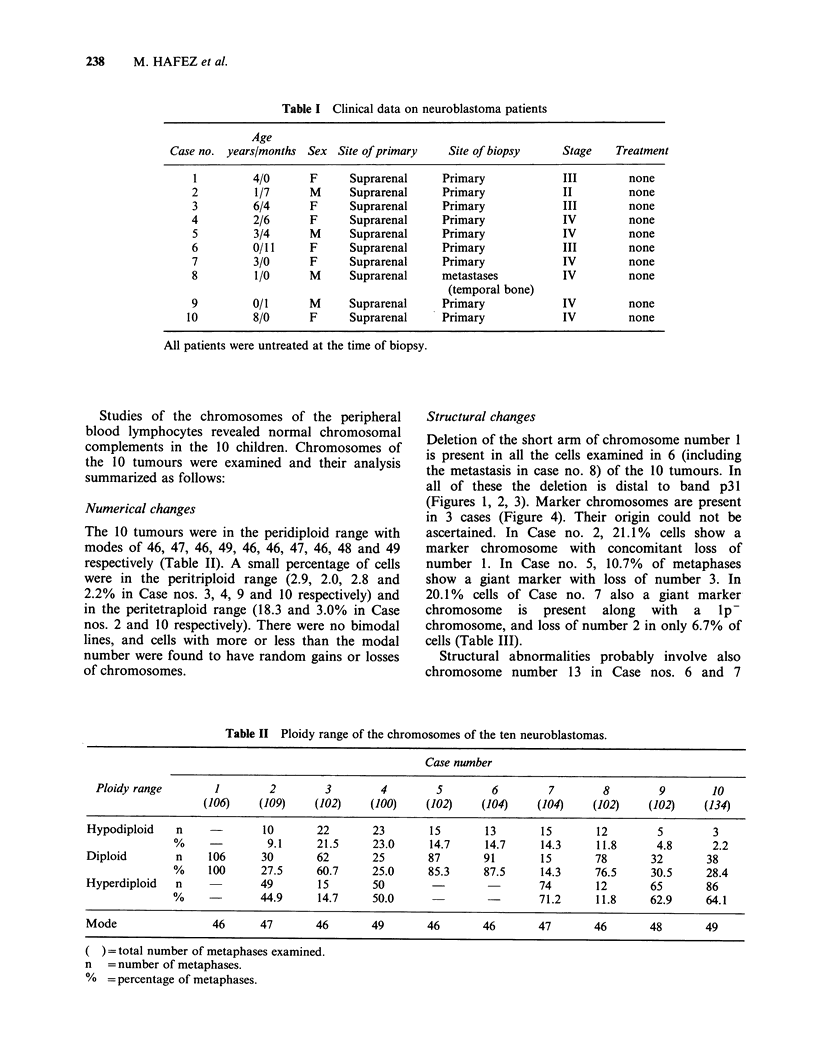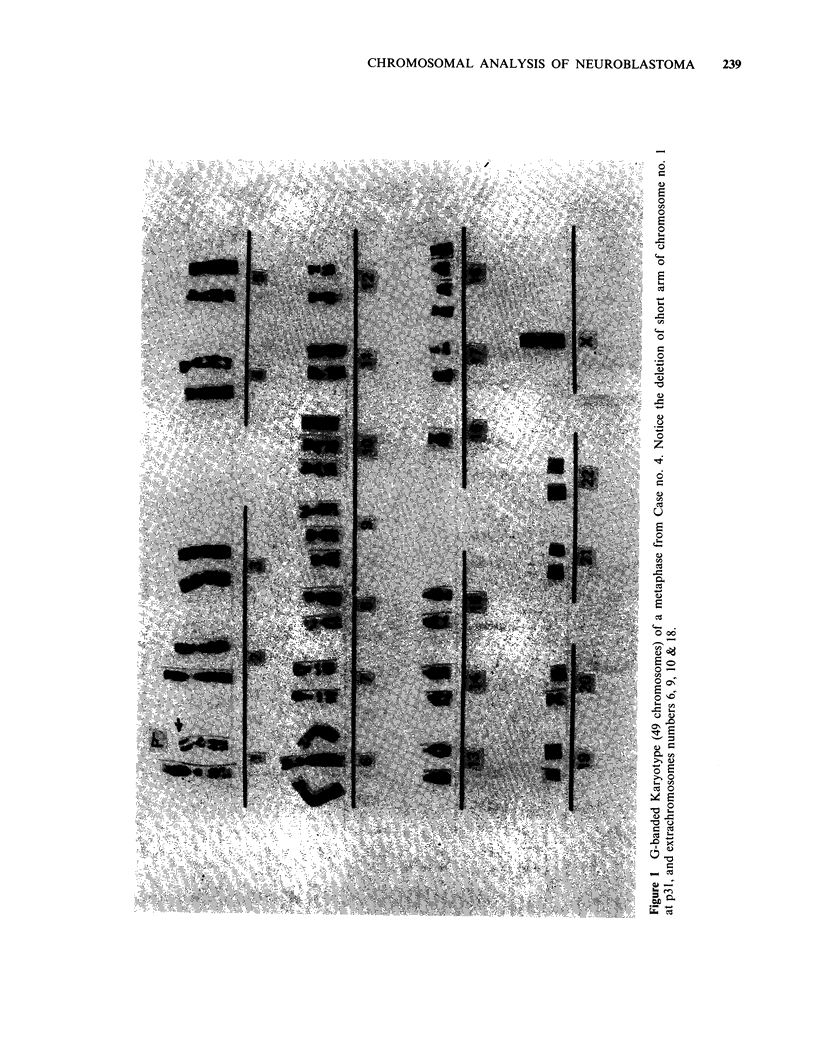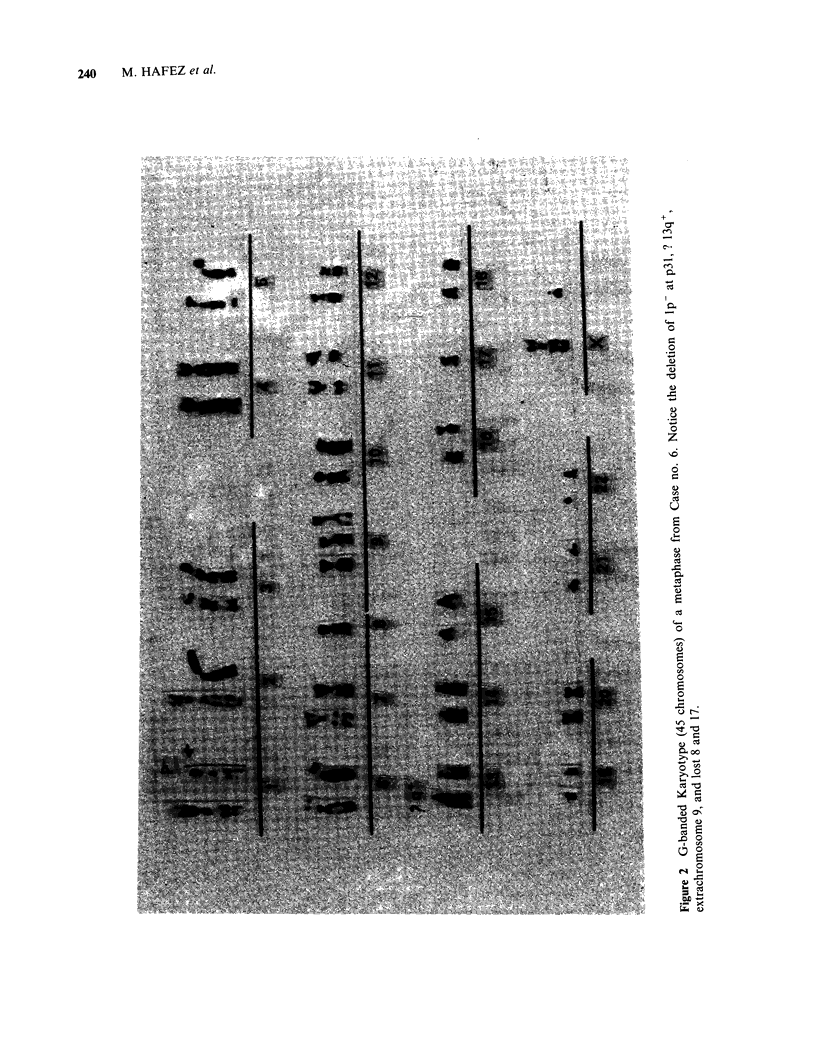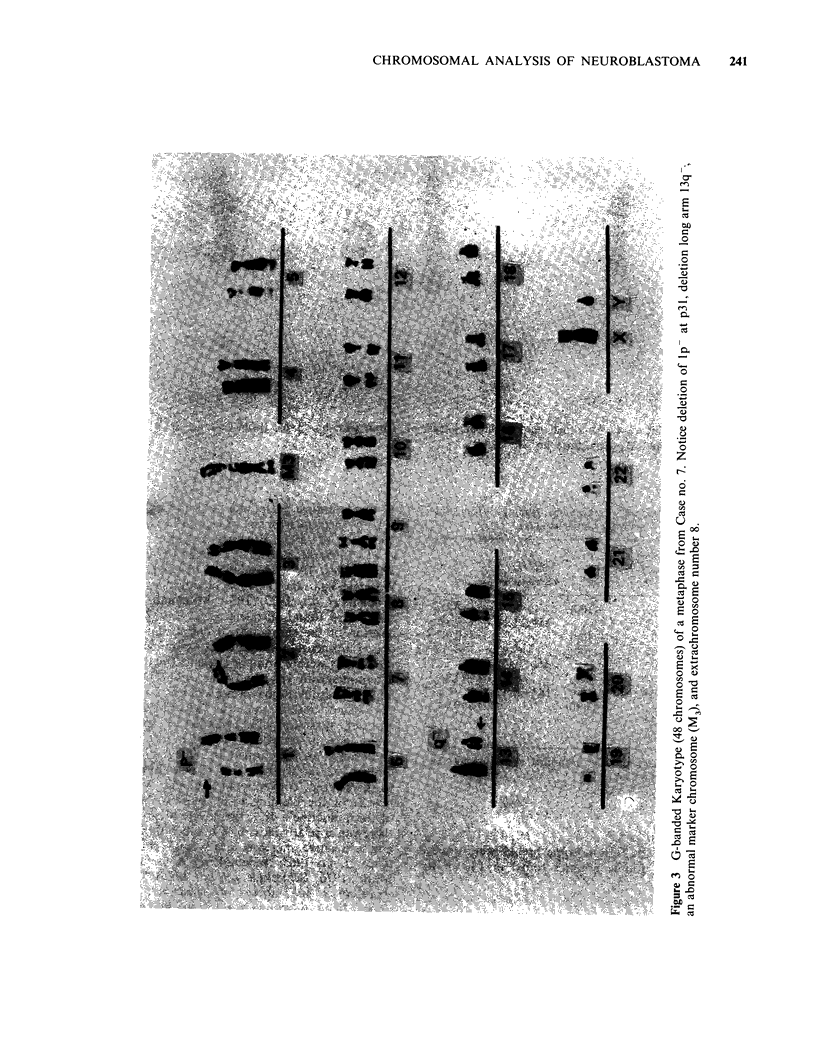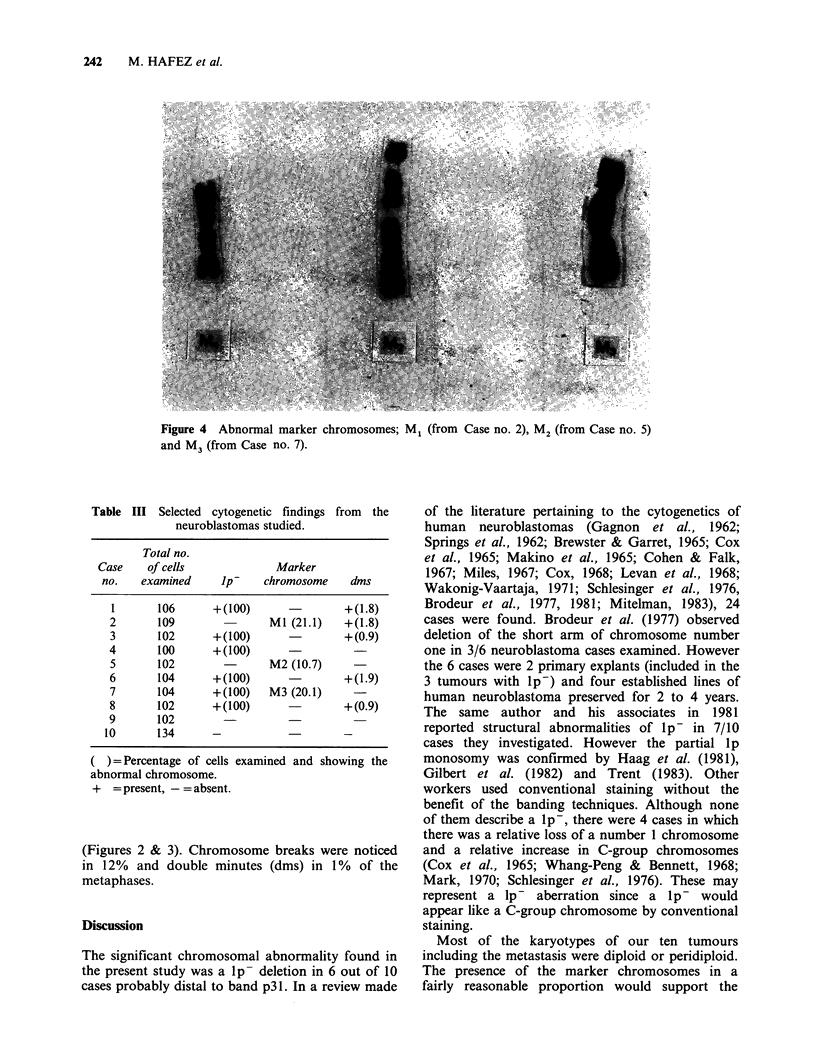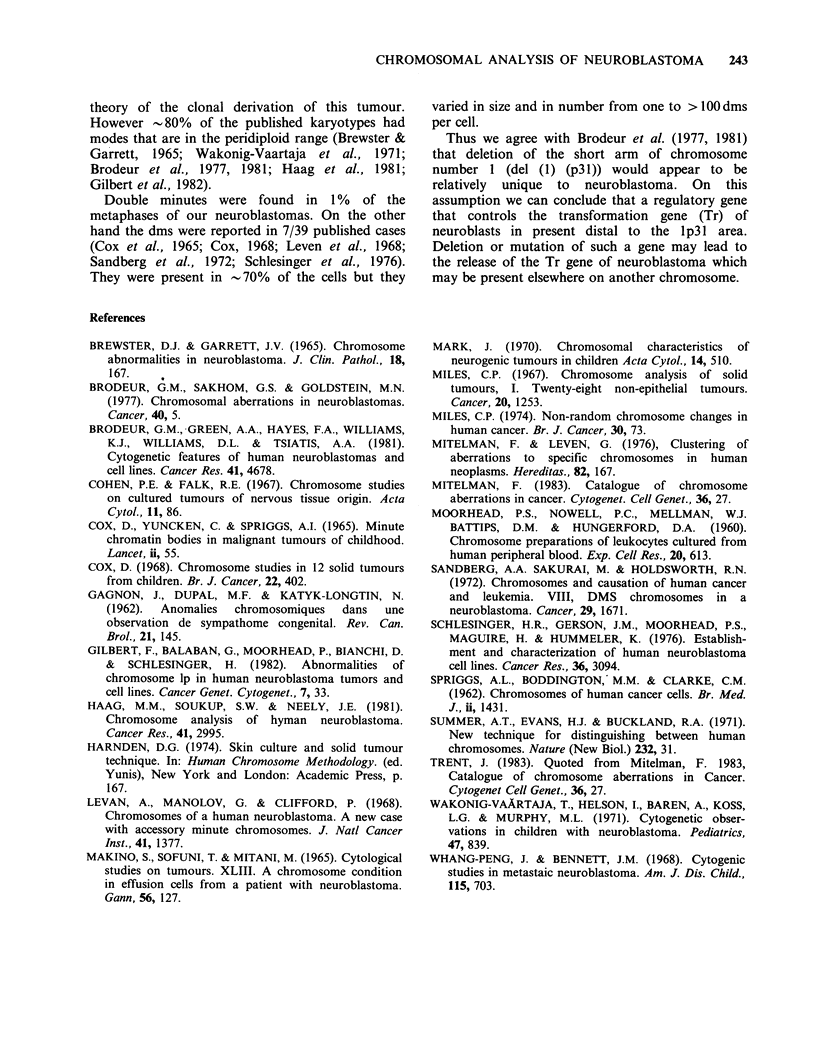# Chromosomal analysis of neuroblastoma.

**DOI:** 10.1038/bjc.1985.34

**Published:** 1985-02

**Authors:** M. Hafez, M. el-Meadday, M. Sheir, Y. Al-Tonbarry, N. Nada, I. el-Desoky

## Abstract

**Images:**


*Br. J. Cancer* (1985), **51**, 237–243

### Chromosomal analysis of neuroblastoma

M. Hafez[1], M. El-Meadday[2], M. Sheir[3], Y. AL-Tonbarry[4], N. Nada[5] & I. EL-Desoky[5]

[1]*Medical Genetics and* [4]*Oncology Units, Pediatrics Department;* [2]*Surgical Oncology and* [3]*Pediatric Surgery Units, Surgery Department; and* [5]*Pathology Department, Faculty of Medicine, University of Mansoura, Egypt.*

**Summary** Ten children with abdominal neuroblastoma were included in the study. Biopsies from the neuroblastomas were taken during surgical operations, nine from the primary tumours and one from a metastasis. Histopathology was done for diagnosis. Chromosomal cultures of neuroblastoma cells and peripheral blood lymphocytes were performed.

The peripheral blood lymphocytes revealed normal chromosomal complements. The 10 tumours were in the peridiploid range with random gains or losses of chromosomes. Deletion of the short arm of chromosome number 1 distal to band p31 occurred in 6 tumours. Other structural changes and giant markers were found. It was concluded that a regulatory gene controlling the transformation gene of neuroblastoma, is present at or distal to lp31.

Neuroblastoma is a malignant tumour arising from the primitive neural crest cells that form the adrenal medulla and sympathetic nervous system. It is one of the most common malignancies of young children. The histologic composition of this tumour is mainly of primitive neuroblast cells.

Analysis of chromosomes in some malignancies such as chronic myelogenous leukaemia and retinoblastoma has been informative. The clustering of aberrations to specific chromosomes in neoplasms indicates that these changes are non-random and possibly related to malignant transformation (Miles 1974; Mitelman & Levan, 1976). The reported chromosome numbers in neuroblastomas vary considerably but are in the peridiploid range (Brewster & Garrett, 1965; Brodeur *et al.*, 1977). Sandberg *et al.* (1972) reported double minute chromosomes in neuroblastoma, while Brodeur *et al.* (1977) could not confirm this. However, these were also found frequently in glial as well as other neurogenic tumours. Some workers reported deletion of the short arm of chromosome number 1 in some of their neuroblastomas (Brodeur *et al.*, 1977, 1981; Haag *et al.*, 1981; Gilbert *et al.*, 1982 and Trent, 1983).

Since there is no previous report of clear and specific chromosomal abnormalities in neuroblastoma, we planned this cytogenetic study in order to search for any specific aberration.

Correspondence: M. Hafez.
Received 9 August 1984; and in revised form 6 November 1984.

### Materials and methods

Ten children with abdominal neuroblastoma were included in the study. The clinical picture and stages are presented in Table I. No child had any congenital anomalies or dysmorphic features. Chromosomal studies were carried out on cultures of peripheral blood lymphocytes from the 10 children. Biopsies from the primary abdominal neuroblastomas in 9 cases and from a metastasis in the temporal bone in one case were taken during surgical operations in the Surgical Oncology and Pediatric Surgery Units, Mansoura University Hospital during the last 4 years (1980–84). Histopathology of tumour specimens was done for diagnosis. Direct preparations from tumour material was carried out (Harnden, 1974). Chromosomal analysis was performed with a modification of the technique of Moorhead *et al.* (1960). Slides were stained by the ASG technique for G-band identification of each chromosome (Sumner *et al.*, 1971). At least 100 cells were counted and 20 photographed for each of the tumour explants and for peripheral blood lymphocytes.

### Results

Histopathologic studies of the 10 tumour specimens revealed the classical picture of neuroblastoma. The tissue comprised primitive neuroblasts, which were rounded, with darkly stained nuclei, prominant nucleoli and scanty cytoplasm. The cells were poorly supported by stroma.

© The Macmillan Press Ltd., 1985

**Table I** Clinical data on neuroblastoma patients

| *Case no.* | *Age years/months* | *Sex* | *Site of primary* | *Site of biopsy* | *Stage* | *Treatment* |
|---|---|---|---|---|---|---|
| 1 | 4/0 | F | Suprarenal | Primary | III | none |
| 2 | 1/7 | M | Suprarenal | Primary | II | none |
| 3 | 6/4 | F | Suprarenal | Primary | III | none |
| 4 | 2/6 | F | Suprarenal | Primary | IV | none |
| 5 | 3/4 | M | Suprarenal | Primary | IV | none |
| 6 | 0/11 | F | Suprarenal | Primary | III | none |
| 7 | 3/0 | F | Suprarenal | Primary | IV | none |
| 8 | 1/0 | M | Suprarenal | metastases (temporal bone) | IV | none |
| 9 | 0/1 | M | Suprarenal | Primary | IV | none |
| 10 | 8/0 | F | Suprarenal | Primary | IV | none |

All patients were untreated at the time of biopsy.

Studies of the chromosomes of the peripheral blood lymphocytes revealed normal chromosomal complements in the 10 children. Chromosomes of the 10 tumours were examined and their analysis summarized as follows:

*Numerical changes*

The 10 tumours were in the peridiploid range with modes of 46, 47, 46, 49, 46, 46, 47, 46, 48 and 49 respectively (Table II). A small percentage of cells were in the peritriploid range (2.9, 2.0, 2.8 and 2.2% in Case nos. 3, 4, 9 and 10 respectively) and in the peritetraploid range (18.3 and 3.0% in Case nos. 2 and 10 respectively). There were no bimodal lines, and cells with more or less than the modal number were found to have random gains or losses of chromosomes.

*Structural changes*

Deletion of the short arm of chromosome number 1 is present in all the cells examined in 6 (including the metastasis in case no. 8) of the 10 tumours. In all of these the deletion is distal to band p31 (Figures 1, 2, 3). Marker chromosomes are present in 3 cases (Figure 4). Their origin could not be ascertained. In Case no. 2, 21.1% cells show a marker chromosome with concomitant loss of number 1. In Case no. 5, 10.7% of metaphases show a giant marker with loss of number 3. In 20.1% cells of Case no. 7 also a giant marker chromosome is present along with a $1p^-$ chromosome, and loss of number 2 in only 6.7% of cells (Table III).

Structural abnormalities probably involve also chromosome number 13 in Case nos. 6 and 7

**Table II** Ploidy range of the chromosomes of the ten neuroblastomas.

| *Ploidy range* | | *Case number* | | | | | | | | | |
|---|---|---|---|---|---|---|---|---|---|---|---|
| | | *1* (*106*) | *2* (*109*) | *3* (*102*) | *4* (*100*) | *5* (*102*) | *6* (*104*) | *7* (*104*) | *8* (*102*) | *9* (*102*) | *10* (*134*) |
| Hypodiploid | n | — | 10 | 22 | 23 | 15 | 13 | 15 | 12 | 5 | 3 |
| | % | — | 9.1 | 21.5 | 23.0 | 14.7 | 14.7 | 14.3 | 11.8 | 4.8 | 2.2 |
| Diploid | n | 106 | 30 | 62 | 25 | 87 | 91 | 15 | 78 | 32 | 38 |
| | % | 100 | 27.5 | 60.7 | 25.0 | 85.3 | 87.5 | 14.3 | 76.5 | 30.5 | 28.4 |
| Hyperdiploid | n | — | 49 | 15 | 50 | — | — | 74 | 12 | 65 | 86 |
| | % | — | 44.9 | 14.7 | 50.0 | — | — | 71.2 | 11.8 | 62.9 | 64.1 |
| Mode | | 46 | 47 | 46 | 49 | 46 | 46 | 47 | 46 | 48 | 49 |

( ) = total number of metaphases examined.
n = number of metaphases.
% = percentage of metaphases.

**Figure 4** Abnormal marker chromosomes; $M_1$ (from Case no. 2), $M_2$ (from Case no. 5) and $M_3$ (from Case no. 7).

**Table III** Selected cytogenetic findings from the neuroblastomas studied.

| *Case no.* | *Total no. of cells examined* | *1p⁻* | *Marker chromosome* | *dms* |
|---|---|---|---|---|
| 1 | 106 | +(100) | — | +(1.8) |
| 2 | 109 | — | M1 (21.1) | +(1.8) |
| 3 | 102 | +(100) | — | +(0.9) |
| 4 | 100 | +(100) | — | — |
| 5 | 102 | — | M2 (10.7) | — |
| 6 | 104 | +(100) | — | +(1.9) |
| 7 | 104 | +(100) | M3 (20.1) | — |
| 8 | 102 | +(100) | — | +(0.9) |
| 9 | 102 | — | — | — |
| 10 | 134 | – | — | – |

( ) = Percentage of cells examined and showing the abnormal chromosome.
+ = present, – = absent.

(Figures 2 & 3). Chromosome breaks were noticed in 12% and double minutes (dms) in 1% of the metaphases.

### Discussion

The significant chromosomal abnormality found in the present study was a $1p^-$ deletion in 6 out of 10 cases probably distal to band p31. In a review made of the literature pertaining to the cytogenetics of human neuroblastomas (Gagnon *et al.*, 1962; Springs *et al.*, 1962; Brewster & Garret, 1965; Cox *et al.*, 1965; Makino *et al.*, 1965; Cohen & Falk, 1967; Miles, 1967; Cox, 1968; Levan *et al.*, 1968; Wakonig-Vaartaja, 1971; Schlesinger *et al.*, 1976, Brodeur *et al.*, 1977, 1981; Mitelman, 1983), 24 cases were found. Brodeur *et al.* (1977) observed deletion of the short arm of chromosome number one in 3/6 neuroblastoma cases examined. However the 6 cases were 2 primary explants (included in the 3 tumours with $1p^-$) and four established lines of human neuroblastoma preserved for 2 to 4 years. The same author and his associates in 1981 reported structural abnormalities of $1p^-$ in 7/10 cases they investigated. However the partial 1p monosomy was confirmed by Haag *et al.* (1981), Gilbert *et al.* (1982) and Trent (1983). Other workers used conventional staining without the benefit of the banding techniques. Although none of them describe a $1p^-$, there were 4 cases in which there was a relative loss of a number 1 chromosome and a relative increase in C-group chromosomes (Cox *et al.*, 1965; Whang-Peng & Bennett, 1968; Mark, 1970; Schlesinger *et al.*, 1976). These may represent a $1p^-$ aberration since a $1p^-$ would appear like a C-group chromosome by conventional staining.

Most of the karyotypes of our ten tumours including the metastasis were diploid or peridiploid. The presence of the marker chromosomes in a fairly reasonable proportion would support the theory of the clonal derivation of this tumour. However ~80% of the published karyotypes had modes that are in the peridiploid range (Brewster & Garrett, 1965; Wakonig-Vaartaja *et al.*, 1971; Brodeur *et al.*, 1977, 1981; Haag *et al.*, 1981; Gilbert *et al.*, 1982).

Double minutes were found in 1% of the metaphases of our neuroblastomas. On the other hand the dms were reported in 7/39 published cases (Cox *et al.*, 1965; Cox, 1968; Leven *et al.*, 1968; Sandberg *et al.*, 1972; Schlesinger *et al.*, 1976). They were present in ~70% of the cells but they varied in size and in number from one to >100 dms per cell.

Thus we agree with Brodeur *et al.* (1977, 1981) that deletion of the short arm of chromosome number 1 (del (1) (p31)) would appear to be relatively unique to neuroblastoma. On this assumption we can conclude that a regulatory gene that controls the transformation gene (Tr) of neuroblasts in present distal to the 1p31 area. Deletion or mutation of such a gene may lead to the release of the Tr gene of neuroblastoma which may be present elsewhere on another chromosome.

### References

BREWSTER, D.J. & GARRETT, J.V. (1965). Chromosome abnormalities in neuroblastoma. *J. Clin. Pathol.*, **18**, 167.

BRODEUR, G.M., SAKHOM, G.S. & GOLDSTEIN, M.N. (1977). Chromosomal aberrations in neuroblastomas. *Cancer*, **40**, 5.

BRODEUR, G.M., GREEN, A.A., HAYES, F.A., WILLIAMS, K.J., WILLIAMS, D.L. & TSIATIS, A.A. (1981). Cytogenetic features of human neuroblastomas and cell lines. *Cancer Res.* **41**, 4678.

COHEN, P.E. & FALK, R.E. (1967). Chromosome studies on cultured tumours of nervous tissue origin. *Acta Cytol.*, **11**, 86.

COX, D., YUNCKEN, C. & SPRIGGS, A.I. (1965). Minute chromatin bodies in malignant tumours of childhood. *Lancet*, **ii**, 55.

COX, D. (1968). Chromosome studies in 12 solid tumours from children. *Br. J. Cancer*, **22**, 402.

GAGNON, J., DUPAL, M.F. & KATYK-LONGTIN, N. (1962). Anomalies chromosomiques dans une observation de sympathome congenital. *Rev. Can. Brol.*, **21**, 145.

GILBERT, F., BALABAN, G., MOORHEAD, P., BIANCHI, D. & SCHLESINGER, H. (1982). Abnormalities of chromosome lp in human neuroblastoma tumors and cell lines. *Cancer Genet. Cytogenet.*, **7**, 33.

HAAG, M.M., SOUKUP, S.W. & NEELY, J.E. (1981). Chromosome analysis of hyman neuroblastoma. *Cancer Res.*, **41**, 2995.

HARNDEN, D.G. (1974). Skin culture and solid tumour technique. In: *Human Chromosome Methodology*. (ed. Yunis), New York and London: Academic Press, p. 167.

LEVAN, A., MANOLOV, G. & CLIFFORD, P. (1968). Chromosomes of a human neuroblastoma. A new case with accessory minute chromosomes. *J. Natl Cancer Inst.*, **41**, 1377.

MAKINO, S., SOFUNI, T. & MITANI, M. (1965). Cytological studies on tumours. XLIII. A chromosome condition in effusion cells from a patient with neuroblastoma. *Gann*, **56**, 127.

MARK, J. (1970). Chromosomal characteristics of neurogenic tumours in children *Acta Cytol.*, **14**, 510.

MILES, C.P. (1967). Chromosome analysis of solid tumours, I. Twenty-eight non-epithelial tumours. *Cancer*, **20**, 1253.

MILES, C.P. (1974). Non-random chromosome changes in human cancer. *Br. J. Cancer*, **30**, 73.

MITELMAN, F. & LEVEN, G. (1976), Clustering of aberrations to specific chromosomes in human neoplasms. *Hereditas.*, **82**, 167.

MITELMAN, F. (1983). Catalogue of chromosome aberrations in cancer. *Cytogenet. Cell Genet.*, **36**, 27.

MOORHEAD, P.S., NOWELL, P.C., MELLMAN, W.J. BATTIPS, D.M. & HUNGERFORD, D.A. (1960). Chromosome preparations of leukocytes cultured from human peripheral blood. *Exp. Cell Res.*, **20**, 613.

SANDBERG, A.A. SAKURAI, M. & HOLDSWORTH, R.N. (1972). Chromosomes and causation of human cancer and leukemia. VIII, DMS chromosomes in a neuroblastoma. *Cancer*, **29**, 1671.

SCHLESINGER, H.R., GERSON, J.M., MOORHEAD, P.S., MAGUIRE, H. & HUMMELER, K. (1976). Establishment and characterization of human neuroblastoma cell lines. *Cancer Res.*, **36**, 3094.

SPRIGGS, A.L., BODDINGTON, M.M. & CLARKE, C.M. (1962). Chromosomes of human cancer cells. *Br. Med. J.*, **ii**, 1431.

SUMMER, A.T., EVANS, H.J. & BUCKLAND, R.A. (1971). New technique for distinguishing between human chromosomes. *Nature* (New Biol.) **232**, 31.

TRENT, J. (1983). Quoted from Mitelman, F. 1983, Catalogue of chromosome aberrations in Cancer. *Cytogenet Cell Genet.*, **36**, 27.

WAKONIG-VAÅRTAJA, T., HELSON, I., BAREN, A., KOSS, L.G. & MURPHY, M.L. (1971). Cytogenetic observations in children with neuroblastoma. *Pediatrics*, **47**, 839.

WHANG-PENG, J. & BENNETT, J.M. (1968). Cytogenic studies in metastaic neuroblastoma. *Am. J. Dis. Child.*, **115**, 703.